# Inverse association between use of broad spectrum penicllin with beta-lactamase inhibitors and prevalence of type 1 diabetes mellitus in Europe

**DOI:** 10.1038/s41598-021-96301-y

**Published:** 2021-08-18

**Authors:** Gábor Ternák, Károly Berényi, Szilárd Kun, Nóra Szigeti, Tamás Decsi, Gábor Sütő, István Wittmann

**Affiliations:** 1 Department of Operational Medicine, University of Pécs Medical School, Pécs, Hungary; 2 Department of Public Health Medicine, University of Pécs Medical School, Pécs, Hungary; 3 2Nd Department of Medicine and Nephrology-Diabetes Center, University of Pécs Medical School, Pacsirta u. 1, 7624 Pécs, Hungary; 4 Department of Paediatrics, University of Pécs Medical School, Pécs, Hungary

**Keywords:** Endocrinology, Medical research

## Abstract

Increasing incidence of type 1 diabetes is supposed to be induced by environmental factors. Microbiome modulated by antibiotics seems to serve as one of the environmental factors which could influence the development of T1DM. Mitochondria, as autochthonous environmental bacteria living in our cells, and other bacteria share many common enzymes including beta-lactamases and it is supported by evidence that some beta-lactamase inhibitors are able to interact with counterpart enzymes. Thus, antibiotics may utilize two different pathways influencing the development of T1DM; one through modulation of microbiome and a second one via the interaction of mitochondrial enzymes. Data of consumption of penicillin (both narrow and broad spectrum) and beta-lactamase inhibitors in 30 European countries were collected from the database of the European Centre for Disease Prevention and Control. These data were correlated with the prevalence reported by the International Diabetes Federation (2019) referring to type 1 diabetes in Europe. No correlation was found between total penicillin consumption or use of broad spectrum penicillin and the prevalence of type 1 diabetes. Nevertheless, broad spectrum penicillin, in combination with beta-lactamase inhibitor, was in inverse correlation with the prevalence of type 1 diabetes (r = − 0.573, p = 0.001). On the other hand, narrow spectrum penicillin was in positive correlation with type 1 diabetes (r = 0.523, p = 0.003). Prevalence of type 1 diabetes showed an inverse correlation with the use of beta-lactamase inhibitors and a positive one with that of narrow spectrum penicillin. Such a detailed analysis has not so far been provided referring to the penicillin group. In the background of this association either microbiomal or direct mitochondrial effects can be supposed.

## Introduction

T1DM is caused by an autoimmune reaction in which the body’s immune system attacks the insulin-producing beta cells of the pancreas. The causes of this destructive process are not fully understood but a likely explanation is that the combination of genetic susceptibility^1^ and environmental triggers, such as viral infections^2^, can initiate the autoimmune reaction. The disease process typically starts at a young age, earlier than 18 years^3,4^. The rate of T1DM has increased conspicuously in most countries by about 3% per annum^5^. The cause of this rise has been only partially identified and a seasonal sinusoidal pattern was described^6^.

Recently, there has been an increasing interest in the effect of intestinal homeostasis on the development of T1DM. Microbial dysbiosis with altered permeability of the gut barrier has been documented in T1DM subjects^7^. Accumulating scientific evidence indicates the probable role of antibiotic-modified microbiome in the development of immunological disturbances leading in this way to T1DM^8–10^.

To examine the possible association between antibiotic consumption patterns and the development of diabetes, a large database of antibiotic consumption (European Centre for Disease Prevention and Control, ECDC database)^11^ was compared with the European data issued by the International Diabetes Federation (IDF) regarding the prevalence of T1DM (patients with diabetes younger than 18 years), as it appeared in the Diabetes Atlas of IDF, 9th edition (2019)^12^.

Another focus of this study was the role of beta-lactamase inhibitors, because they, such as clavulanic acid and sulbactam, are prescribed for patients in combination with penicillin, for instance ampicillin. Interestingly, 18 different types of beta-lactamase-like metalloproteins have been detected in human cells in the mitochondria^13^. Moreover, using in vitro experiments, an inhibition of some of these mitochondrial enzymes was proved by the beta-lactamase blocker sulbactame^14^. These data suggest that beta-lactamase inhibitors may have a direct effect on the human body.

It is suspected that penicillin and beta-lactamase consumption patterns of different European countries might be reflected by the prevalence figures of T1DM.

## Materials and methods

### Databases

Data of cumulative yearly consumptions of penicillin (J01C) and different penicillin subgroups [broad spectrum, penicillinase sensitive penicillin (J01CA), broad spectrum penicillin combined with penicillinase (beta-lactamase) inhibitors (J01CR), narrow spectrum, penicillinase sensitive penicillin (J01CE) and narrow spectrum penicillinase resistant penicillin (J01CF)] in 30 European countries were obtained from databases of the European Centre for Disease Prevention and Control (ECDC)^11^. Combined groups were also calculated: that of total broad spectrum penicillin (J01CA + J01CR) and total narrow spectrum penicillin (J01CE + J01CF). Three time-periods were observed: 1997–2007; 2008–2018 and the combination of these (1997–2018). The total amount of antibiotic use was expressed in Defined Daily Dose (DID) (/1000 population/day). Average yearly consumption data of penicillin and the above mentioned penicillin subgroups were calculated and expressed in percentage (%) of the relative average share of the total amount (in DID). Antibiotic consumption was estimated at ATC levels 3 and 4.

Prevalence of T1DM in the 0–19-year age group in certain countries were extracted from the Diabetes Atlas 2019 of the International Diabetes Federation^14^ and was calculated for 100,000 inhabitants. Principally, we have got the same results using the Diabetes Atlas 2017, estimation method and limitations of which are described elsewhere^15^.

### Statistics

Spearman correlation was used to calculate the association between cumulative yearly consumption of the penicillin subgroups and prevalence of childhood and adolescent T1DM, as distribution of data of T1DM prevalence and most of the penicillin subgroup consumptions were not normal according to Kolmogorov–Smirnov’s test. A correlation was considered as signifcant in case *p* value was < 0.05.

## Results

Total antibiotic use and cumulative yearly consumptions of penicillin and penicillin subgroups of 30 European countries for periods 1997–2007; 2008–2018 and 1997–2018 are shown in Tables 1, 2, 3, respectively. Table 3 also contains data of T1DM prevalence in the 0–19-year age group.

**Table 1:** Cumulative yearly penicillin consumption (ECDC database 1997–2007).

	Total antibiotic consumption^a^	Total penicillin^b^	Broad spectrum, penicillinase sensitive penicillin^c^	Broad spectrum penicillin combined with penicillinase inhibitors^d^	Total broad spectrum penicillin^e^	Narrow spectrum, penicillinase sensitive penicillin^f^	Narrow spectrum penicillinase resistant penicillin^g^	Total narrow spectrum penicillin^h^
Country	(DID 100%)	(%)	(%)	(%)	(%)	(%)	(%)	(%)
Austria	11.760	32.398	7.262	15.102	22.364	9.940	0.085	12.959
Belgium	21.350	33.068	12.693	18.436	31.129	0.723	1.208	10.909
Bulgaria	17.200	44.444	28.663	5.820	34.483	9.683	2.131	7.384
Croatia	19.743	41.606	16.223	17.359	33.581	7.742	0.325	8.668
Cyprus	27.900	36.380	16.416	19.534	35.950	0.430	0.011	13.620
Czech Rep	13.513	38.071	11.841	0.614	12.455	14.154	0.649	11.347
Denmark	13.109	61.484	16.912	0.328	17.240	38.485	5.874	3.494
Estonia	10.686	34.275	24.098	4.527	28.624	3.021	0.468	7.872
Finland	17.418	29.750	15.527	3.805	19.332	10.650	0.553	5.668
France	26.136	38.330	22.748	12.539	35.287	0.761	1.718	8.355
Germany	12.564	29.739	31.949	2.199	34.149	11.517	0.144	9.847
Greece	28.627	25.850	12.946	10.361	23.307	2.543	0.021	9.844
Hungary	16.400	37.805	13.232	18.091	31.323	6.421	0.000	9.707
Iceland	20.182	48.559	18.536	9.013	27.549	14.394	6.315	3.676
Ireland	16.870	41.968	15.649	16.366	32.015	4.813	5.033	5.625
Italy	21.733	38.753	20.900	17.648	38.548	0.112	0.097	14.841
Latvia	10.200	37.843	26.745	9.510	36.255	1.608	0.039	10.039
Lithuania	20.900	57.416	25.837	4.928	30.766	24.809	1.866	5.478
Luxembourg	23.664	31.322	13.150	16.738	29.889	0.668	0.818	10.058
Malta	15.000	39.333	6.200	31.933	38.133	0.533	0.600	11.333
Netherlands	9.064	31.795	13.990	9.458	23.448	5.263	2.908	10.080
Norway	15.313	41.306	11.600	0.033	11.632	13.725	2.620	3.877
Poland	16.789	34.613	27.034	6.380	33.414	3.898	0.231	7.432
Portugal	18.891	38.258	14.584	21.660	36.244	0.222	3.190	15.943
Slovakia	22.833	48.224	15.260	12.681	27.941	20.175	0.160	8.112
Slovenia	14.691	51.794	14.387	19.808	34.195	16.364	0.965	10.229
Spain	15.809	46.751	20.109	24.313	44.422	0.740	1.512	14.175
Sweden	14.873	45.354	6.784	1.021	7.805	29.658	7.689	6.754

Penicillin consumption (1997–2007), expressed in % of the total consumption (SUM) in DID, according to the ECDC (European Centre for Disease Prevention and Control) database.

ATC code: ^a^J01; ^b^J01C; ^c^J01CA; ^d^J01CR; ^e^J01CA + J01CR; ^f^J01CE; ^g^J01CF; ^h^J01CE + J01CF.

**Table 2:** Cumulative yearly penicillin consumption (ECDC database 2008–2018).

	Total antibiotic consumption^a^	Total penicillin^b^	Broad spectrum, penicillinase sensitive penicillin^c^	Broad spectrum penicillin combined with penicillinase inhibitors^d^	Total broad spectrum penicillin^e^	Narrow spectrum, penicillinase sensitive penicillin^f^	Narrow spectrum penicillinase resistant penicillin^g^	Total narrow spectrum penicillin^h^
Country	(DID 100%)	(%)	(%)	(%)	(%)	(%)	(%)	(%)
Austria	12.45	38.61	6.48	25.52	32.00	6.76	0.07	6.82
Belgium	22.52	46.10	21.28	23.27	44.55	0.21	1.13	1.34
Bulgaria	17.54	31.83	19.28	11.57	30.84	1.36	0.00	1.36
Croatia	17.86	42.57	11.74	26.46	38.19	4.32	0.05	4.37
Cyprus	26.77	34.94	10.39	24.35	34.74	0.32	0.09	0.41
Czech Rep	16.51	35.88	7.16	16.67	23.83	11.61	0.23	11.85
Denmark	15.24	63.09	20.54	4.21	24.75	29.46	8.73	38.19
Estonia	10.23	31.73	17.36	12.38	29.74	2.05	0.01	2.06
Finland	16.16	29.75	16.24	5.27	21.51	7.87	0.22	8.09
France	23.62	50.54	29.30	19.30	48.60	0.67	1.17	1.84
Germany	13.23	25.15	16.72	2.56	19.28	5.87	0.08	5.94
Greece	32.22	29.26	13.66	14.70	28.35	0.87	0.00	0.87
Hungary	13.65	33.95	7.03	24.39	31.42	2.61	0.00	2.61
Iceland	18.76	47.87	16.45	14.36	30.81	11.66	5.56	17.21
Ireland	19.51	47.34	14.22	20.87	35.09	6.24	6.73	12.98
Italy	22.23	45.40	12.90	31.85	44.75	0.00	0.04	0.04
Latvia	10.75	38.21	26.26	11.51	37.77	0.64	0.01	0.65
Lithuania	14.97	45.78	33.26	9.37	42.63	3.27	0.04	3.31
Luxembourg	23.35	37.14	12.95	22.53	35.48	0.18	0.89	1.07
Malta	19.18	33.08	2.94	30.15	33.09	0.38	0.24	0.62
Netherlands	9.62	32.42	13.47	11.38	24.85	2.82	4.36	7.17
Norway	15.28	40.04	13.73	0.04	13.76	22.09	4.00	26.10
Poland	20.39	33.08	17.11	14.76	31.87	1.07	0.03	1.10
Portugal	17.79	46.70	9.35	34.06	43.41	0.10	3.24	3.34
Romania	26.14	43.56	16.74	21.42	38.16	2.86	2.41	5.27
Slovakia	20.33	30.50	5.70	17.64	23.34	6.67	0.00	6.67
Slovenia	11.68	59.92	19.39	24.26	43.66	15.04	1.26	16.30
Spain	18.72	54.78	21.07	31.94	53.01	0.45	1.09	1.54
Sweden	12.74	49.89	8.54	1.63	10.17	27.86	11.67	39.53
UK	16.945	38.412	20.719	4.614	25.333	4.764	8.160	12.923

Penicillin consumption (2008–2018), expressed in % of the total consumption (SUM) in DID, according to the ECDC (European Centre for Disease Prevention and Control) database.

ATC code: ^a^J01; ^b^J01C; ^c^J01CA; ^d^J01CR; ^e^J01CA + J01CR; ^f^J01CE; ^g^J01CF; ^h^J01CE + J01CF.

**Table 3:** Prevalence of T1DM for 0–19 years and penicillin consumption (ECDC database 1997–2018).

Country	T1DM prevalence (0–19 years)	Cumulative yearly antibiotic consumption (ECDC database 1997–2018)								
	(/100,000 inhabitants)	Total antibiotic consumption^a^		Total penicillin^b^	Broad spectrum, penicillinase sensitive penicillin^c^	Broad spectrum penicillin combined with penicillinase inhibitors^d^	Total broad spectrum penicillin^e^	Narrow spectrum, penicillinase sensitive penicillin^f^	Narrow spectrum penicillinase resistant penicillin^g^	Total narrow spectrum penicillin^h^
		(DID 100%)		(%)	(%)	(%)	(%)	(%)	(%)	(%)
Austria	32.89	12.124		35.742	6.920	20.700	27.540	8.228	0.074	8.303
Belgium	36.94	21.962		40.069	17.300	21.030	38.310	0.437	1.166	1.603
Bulgaria	15.54	17.385		37.446	23.450	9.000	32.460	5.065	0.095	5.160
Croatia	32.37	18.594		42.187	13.580	22.700	36.280	5.733	0.161	5.895
Cyprus	33.02	26.946		35.170	11.340	23.580	34.930	0.337	0.094	0.430
Czech Rep	38.39	15.013		36.714	6.660	13.030	22.360	12.756	0.431	13.187
Denmark	54.35	14.182		62.588	19.010	2.510	21.340	33.527	7.501	41.028
Estonia	46.11	10.406		32.877	20.290	9.000	29.300	2.439	0.061	2.500
Finland	130.92	16.791		29.751	15.870	4.500	20.370	9.228	0.395	9.623
France	41.833	24.877		44.126	26.850	15.740	41.600	0.716	1.458	2.173
Germany	39.53	12.895		27.388	16.650	2.380	19.030	8.497	0.109	8.605
Greece	29.87	30.423		27.814	13.440	12.790	26.170	1.621	0.011	1.632
Hungary	36.46	14.962		35.964	10.260	21.100	31.360	4.589	0.000	4.589
Iceland	36.17	19.473		48.226	17.470	11.580	29.060	12.987	5.950	18.937
Ireland	66.21	18.252		44.978	14.840	18.880	33.730	5.051	5.984	11.035
Italy	26.4	22.005		42.445	16.450	25.530	41.990	0.050	0.068	0.118
Latvia	13.5	10.581		38.098	26.400	10.900	37.310	0.933	0.017	0.950
Lithuania	51.18	15.885		48.135	31.750	8.460	40.220	7.627	0.411	8.038
Luxembourg	35.75	23.009		34.966	13.420	20.040	33.330	0.422	0.871	1.293
Malta	43.18	18.833		33.495	3.150	30.260	33.420	0.389	0.265	0.655
Netherlands	42.74	9.341		32.117	13.720	10.440	24.170	4.063	3.654	7.717
Norway	70.66	15.295		40.571	12.830	0.000	12.680	24.157	3.420	27.577
Poland	33.16	18.770		33.697	20.940	12.030	32.330	2.206	0.112	2.318
Portugal	24.69	18.341		42.354	11.900	27.670	39.570	0.161	3.214	3.375
Romania	14.75	24.144		47.170	18.120	23.190	41.320	3.101	2.604	5.706
Slovakia	46.9	21.516		39.408	10.500	15.140	25.620	13.459	0.080	13.539
Slovenia	28.37	13.186		55.395	16.600	21.780	23.520	15.662	1.096	16.758
Spain	33.07	17.264		51.106	20.620	28.440	49.070	0.579	1.282	1.861
Sweden	85.09	13.805		47.448	7.590	1.300	8.170	28.679	9.526	38.205
UK	57.78	15.259		38.397	21.370	4.950	26.320	4.859	7.149	12.008

Prevalence data of T1DM for 0–19 years calculated for 100,000 inhabitants/country (Diabetes Atlas edition 9) compared to penicillin consumption (1997–2018), expressed in % of the total consumption (SUM) in DID, according to the ECDC (European Centre for Disease Prevention and Control) database.

ATC code: ^a^J01; ^b^J01C; ^c^J01CA; ^d^J01CR; ^e^J01CA + J01CR; ^f^J01CE; ^g^J01CF; ^h^J01CE + J01CF.

Results of the correlations of T1DM prevalence and use of penicillin subgroups are represented in Table 4. Correlations of T1DM prevalence in 2019 were investigated with penicillin use in the three mentioned time periods (1997–2007, 2008–2018 and 1997–2018) separately. Similar results were obtained along these different periods, and results similar to the IDF Diabetes Atlas 2019, using the IDF Diabetes Atlas 2017, as well (data not shown).

**Table 4:** Correlations of antibiotic consumption and prevalences of T1DM (0–19 years).

Subgroups of penicillin	Period		
	1997–2007	2008–2018	1997–2018
Total penicillin^a^	0.122	− 0.027	− 0.028
Broad spectrum, penicillinase sensitive penicillin^b^	− 0.228	− 0.018	− 0.108
Broad spectrum penicillin combined with penicillinase inhibitors^c^	− **0.501**	− **0.558**	− **0.573**
Total broad spectrum penicillin^d^	− **0.583**	− **0.563**	− **0.507**
Narrow spectrum, penicillinase sensitive penicillin^e^	**0.468**	**0.507**	**0.465**
Narrow spectrum penicillinase resistant penicillin^f^	**0.395**	**0.382**	**0.431**
Total narrow spectrum penicillin^g^	**0.461**	**0.532**	**0.523**

ATC code: ^a^J01C; ^b^J01CA; ^c^J01CR; ^d^J01CA + J01CR; ^e^J01CE; ^f^J01CF; ^g^J01CE + J01CF.

*r* values of significant correlations are given in boldface.

**Figure 1:**
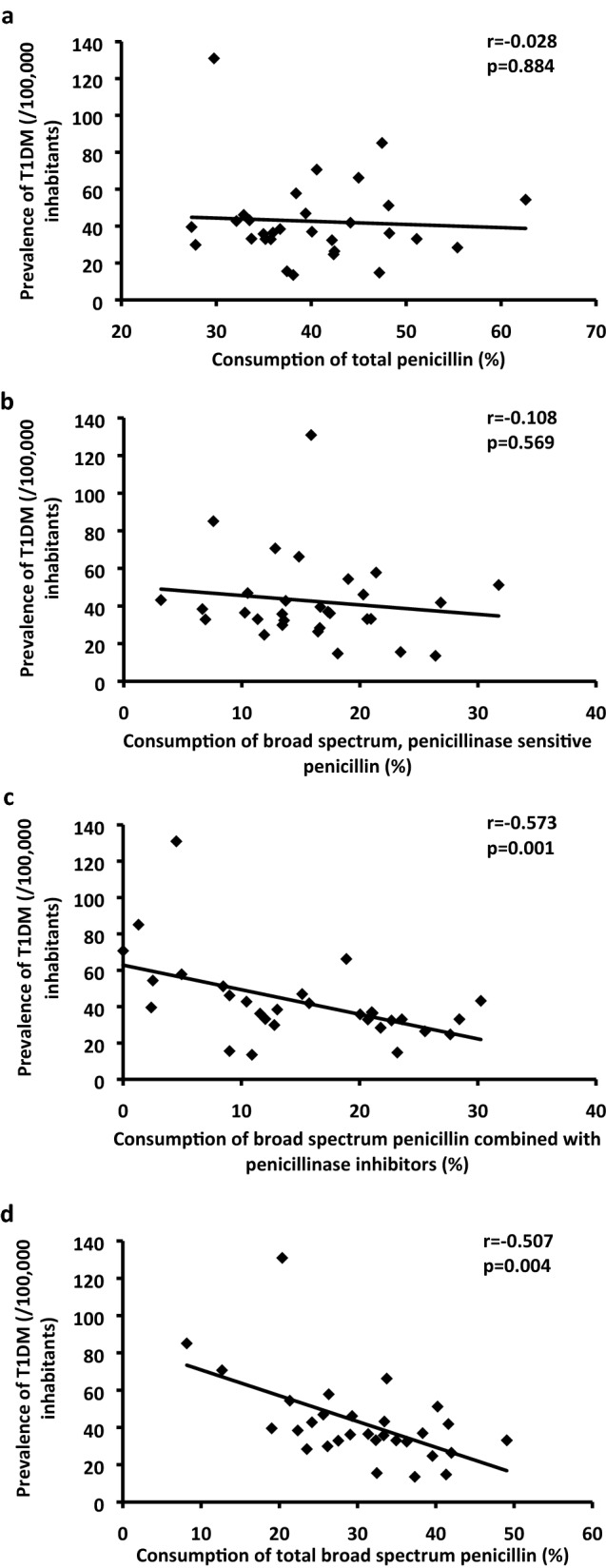
Association of consumption of total penicillin (**a**), broad spectrum, penicillinase sensitive penicillin (**b**), broad spectrum penicillin combined with penicillinase inhibitors (**c**) and total broad spectrum penicillin (**d**) with prevalence of T1DM (0–19 years). Total broad spectrum penicillin represents broad spectrum, penicillinase sensitive penicillin + broad spectrum penicillin combined with penicillinase inhibitors.

No association was observed with total penicillin use (Fig. 1a).

Further analysing the penicillin group at ATC level 4, no correlation was found with broad spectrum, penicillinase sensitive penicillin use (Fig. 1b), while strong negative associations were observed with the use of broad spectrum penicillin combined with penicillinase (beta-lactamase) inhibitors (Fig. 1c) and with total broad spectrum penicillin consumption (Fig. 1d).

**Figure 2:**
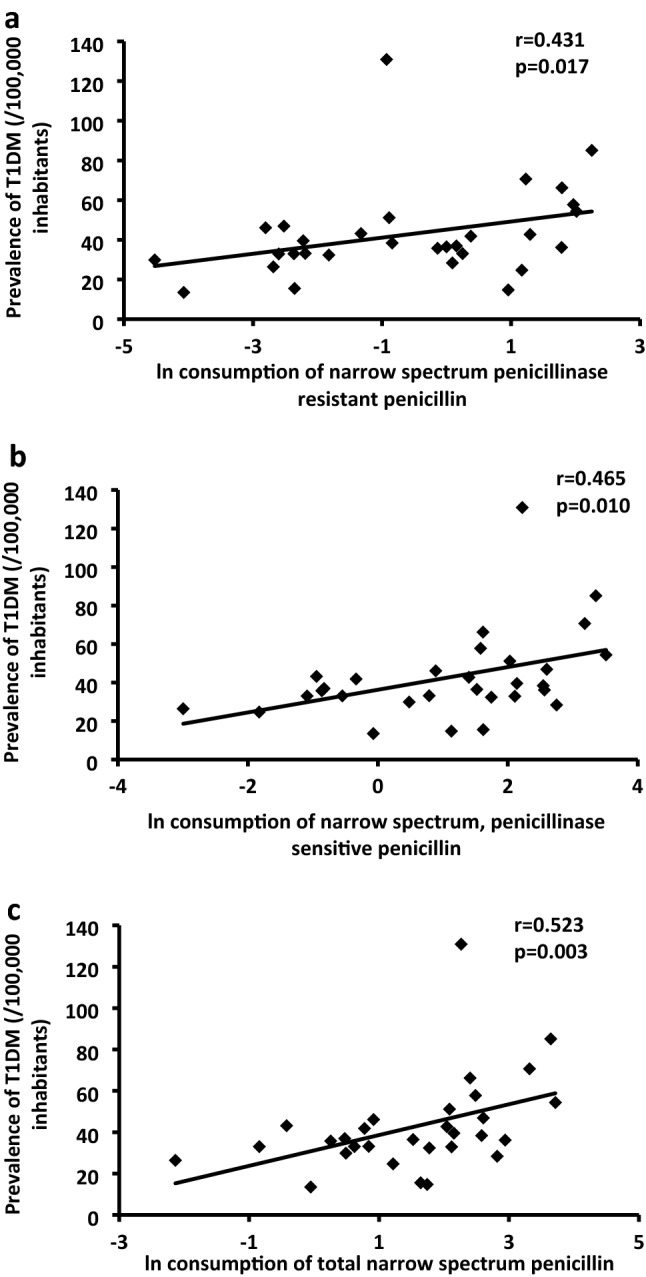
Association of consumption of narrow spectrum, penicillinase sensitive penicillin (**a**), narrow spectrum penicillinase resistant penicillin (**b**) and total narrow spectrum penicillin (**c**) with prevalence of T1DM (0–19 years). Total narrow spectrum penicillin represents narrow spectrum, penicillinase sensitive penicillin + narrow spectrum penicillinase resistant penicillin. Antibiotic consumptions were expressed in natural logarithm, regarding their distribution.

On the contrary, T1DM prevalence showed strong positive correlations with consumption of narrow spectrum, penicillinase sensitive penicillin (Fig. 2a), narrow spectrum penicillinase resistant penicillin (Fig. 2b) and with total narrow spectrum penicillin use (Fig. 2c).

## Discussion

The main findings of this study are, that (1) using broad spectrum penicillins is not associated with the prevalence of T1DM, (2) use of broad spectrum penicillins with beta-lactamase inhibitors is in inverse correlation with the prevalence of T1DM, (3) a positive correlation can be found between the use of both penicillinase sensitive and resistant narrow spectrum penicillins as well as the prevalence of T1DM.

Our results strongly support the idea that penicillin consumption patterns in different countries may influence the epidemiology of T1DM. The unsolved “mystery” of the high incidence of T1DM in Scandinavian countries might be associated with the consumption of penicillins. The prevalence of T1DM in Denmark, Finland, Norway and Sweden calculated for 100,000 inhabitants is the highest in Europe, with 54.35, 130.92, 70.66, and 85.09 cases, respectively. In contrast, a quite high narrow spectrum penicillin consumption of 41.028, 9.623, 27.577, 38.205%, and by far the lowest use of broad spectrum penicillin combined with beta-lactamase inhibitor, 2.510, 4.500, 0.000, 1.300% was observed in these countries, respectively. The consumption of a relatively high rate of narrow spectrum penicillin and a very low rate of beta-lactamase inhibitor combinations may contribute to the high prevalence of T1DM in this region of Europe.

In our study there was no correlation between the prevalence of T1DM and the use of broad spectrum penicillin neither in the 1997–2007, nor in 2008–2018 nor in the whole of 1997–2018 period (Table 4). On the contrary, significant inverse associations could be observed between the prevalence of T1DM and the consumption of broad spectrum penicillin combined with beta-lactamase inhibitor in all three observational periods (Table 4). Interestingly, narrow spectrum penicillin clearly shows a positive correlation with the prevalence of T1DM. These facts suggest that not penicillin itself, but the beta-lactamase inhibitors are able to result in an inverse correlation between the prevalence of T1DM and the consumption of different types of penicillin products.

Seasonality of community antibiotic use is a well-known phenomenon with an especially clear picture in the case of penicillins. Data of antibiotic sales extracted from the International Medical Services Health’s Xponent database showed for the United States in the period between 1999 and 2007 at least a three-fold higher number of prescriptions of aminopenicillins in January than in July^16^. Similarly, analysis of about 98 million outpatient antibiotic prescriptions in the United States between 2013 and 2015 showed that amoxicillin with clavulanate was 78% (95% confidence intervals: 68% to 129%) more likely to be prescribed in February than in September^17^. Report on outpatient antibiotic use in more than one million Swiss adults showed around 3 defined daily doses (DDDs) of amoxicillin use (with or without clavulanate) per 1000 inhabitants per day in August–September as opposed to about 5 DDDs in the period of December to March^18^. It can be assumed reasonably that the above-outlined seasonal peaks in the use of antibiotics may originate from prescriptions aimed to treat upper respiratory infections that may well be of viral origin in the cold and influenza season.

Seasonality in the manifestation of T1DM was consequently reported in children living in several European countries. In a three-year-long Dutch study including 676 children diagnosed with T1DM per year, the annual incidence rates per hundred thousand children were 6.6 and 6.4 in winter as opposed to 4.9 in spring and 5.4 in summer (p < 0.03)^19^. In 5422 children diagnosed with T1DM in Romania between 1996 and 2015, the maximum monthly incidence value was recorded in January (more than 450) as opposed to the minimum value recorded in June (around 300, p < 0.001)^20^. In 2174 Polish children diagnosed with T1DM between 2010 and 2014, the odds of diagnosis being made between June and August was 57% (95% confidence intervals: 51% to 67%, p < 0.0001) of the odds of diagnosing the disease between December and February^21^. In a multicentre study summarising data obtained in 23,063 diabetic children at 48 European centres, nearly 10% of the patients were diagnosed in January as compared to 7% diagnosed in June (p < 0.04)^22^.

T1DM is more prevalent in countries preferring the use of more antibiotics and vaccinations and improving hygiene standards, decreasing in these way the incidence of infections. This is the so-called “hygiene hypothesis”^23^. In a recent study, in The Environmental Determinants of Diabetes in the Young^24^ HLA genotyped high risk newborns from the high risk countries (Finland and Sweden), and also from Germany and the USA were investigated. In reports of early life (3 months and 4 years of age) antibiotic use and development of autoimmunity were assessed. No association between the use of penicillin or beta-lactamase plus penicillin and beta-cell autoimmunity (insulin autoantibody, glutamic acid decarboxylase autoantibody, tyrosine phosphatase IA-2 autoantibody) could be found. We also proved that total penicillin use is not in correlation with the prevalence of T1DM, probably due to the bidirectional effect of the increase with narrow and decrease with broad spectrum penicillin with beta-lactamase. In TEDDY they did not separate narrow spectrum penicillins as a group, and TEDDY did not use the hard end point of the overt T1DM either, what we, however, implicated. Moreover, they rather targeted the surrogate endpoints of immunological positivity.

Correlations obtained in our study do not strictly reveal causative association, but they raise two possible explanations for these results. On the one hand, antibiotics induced changes of the microbiome, and on the other, direct interactions of beta-lactamase inhibitors with the human beta-lactamase enzymes could be supposed to be in the background of this phenomenon. We are currently investigating the correlations with consumption of other antibiotics, as well.

Regarding microbiome, in an animal model of T1DM (non-obese diabetic, NOD-mouse) in the early postnatal period, vancomycin treated animals presented a lower rate of diabetes and one microbe of the gut became dominant^25^. Nevertheless, in the same NOD-model, vancomycin and a combined antibiotic treatment using streptomycin, colistin, and ampicillin increased the incidence of T1DM^26^. Moreover, transplantation of microbiome of NOD to diabetes resistant mice-induced insulitis^27^, however, germ-free NOD-mice attest metabolic and immunologic disturbances^28^. Another experiment on the mouse-model revealed that early postnatal, low dosage of penicillin treatment caused microbiota perturbation, which was long-lasting, resulting in metabolic changes of the host^29^. Summarizing the animal-based data, they are contradictory regarding gut microbiome and antibiotics and T1DM. In humans, more and more observational studies and several review papers point at bacterial changes in prediabetes and in overt T1DM, and the development of leaky gut leading to the dysregulation of the immune system resulting in beta-cell destruction^30–33^. All these results strongly suggest an association between microbiome and T1DM, but there is no answer to whether all these changes are causes or consequences (the chicken-egg question). Moreover, there is no convincing evidence supporting the role of antibiotic therapy in the development of T1DM.

Regarding beta-lactamase hypothesis, it is interesting that mitochondria of the human cells show some bacterial features supporting the hypothesis assuming that during eukaryogenesis the fermentative, anaerobic host archaeon interacted with the aerobic, organotrophic bacterial partner, which became endosymbiont developing the intracellular mitochondria^34^. Thus, human mitochondria express more than a dozen of different types of beta-lactamase enzymes characteristic of bacteria^13^, of which some are inhibited by sulbactame^14^. All these beta-lactamase enzymes are zinc containing proteins, which metal is necessary for insulin biosynthesis and storage in the beta cells. One of these beta-lactamase enzymes is a human mitochondrial endoribonuclease (LACTB2), which is essential for the functioning of mitochondria^35^. Moreover, LACTB2 regulates mitochondrial fat metabolism by organizing mitochondrial membrane and regulates complex I being a member of the mitochondrial electron transport chain^36^. All these data taken into consideration, it can be hypothesized that beta-lactamase inhibitors may have a direct effect on human cells preventing or slowing down the processes leading to T1DM, ever, direct evidence is lacking.

The strengths of this study are the Europe-wide nature, the clear significance of associations, and long exposure to antibiotic treatment (1997–2018). Our cohort consisted of whole population data. We assume that the parental exposition (pregestational, gestational as well) to different types of stressors including antibiotic treatment may affect the development of T1DM in children. Furthermore, our population-wide data involve not only cases with genetically determined high risk but also incidental T1DM patients. This approach provides a broader view on risk factors of a population, particularly referring to the role of antibiotic consumption developing T1DM. Other studies were also performed to clarify the role of environmental factors in the development of T1DM. The TEDDY study was a prospective 6 centre investigation involving Europe and the United States focusing on children younger than four years with a risk of T1DM due to family involvement or carrying high-risk HLA-DR, DQ genotypes^37^. Consequently, the population studied had a genetically determined risk to develop T1DM, and those who had an incident of T1DM were not involved in this study. This may explain why there was no association between antibiotic consumption and the development of T1DM in the TEDDY programme.

The main limitations are the retrospective approach of the study, and that the antibiotic consumption in the ECDC database is characteristic for the overall population of the countries, not only of those who are younger than 18 years of age.

Concluding, use of beta-lactamase inhibitors seem to be in inverse, while that of narrow spectrum penicillin in positive association with T1DM.

Further studies are urgently needed to verify the possible causative associations between T1DM and beta-lactamase inhibitors in order to introduce these results in clinical routine aiming to prevent further escalation of the prevalence of T1DM.

## Abbreviations

ECDC: European Centre for disease prevention and control
IDF: International diabetes federation
T1DM: Type 1 diabetes
TEDDY: The environmental determinants of diabetes in the young
